# A case of septic shock due to delayed diagnosis of *Cryptosporidium* infection after liver transplantation

**DOI:** 10.1186/s12879-023-08252-6

**Published:** 2023-04-26

**Authors:** Xin Tie, Zhongwei Zhang, Ran Zhou, Yi Li, Jinmei Xu, Wanhong Yin

**Affiliations:** 1 Department of Critical Care Medicine, West China Hospital, Sichuan University, Chengdu, 610041 China; 2 Department of Critical Care Medicine, Sichuan Provincial Corps Hospital of the Chinese People’s Armed Police Force, Leshan, 614700 China

**Keywords:** *Cryptosporidium*, Liver Transplant, Immunosuppression, Diarrhea, Diagnosis

## Abstract

**Background:**

*Cryptosporidium* is recognized as a significant pathogen of diarrhea disease in immunocompromised hosts, and studies have shown that *Cryptosporidium* infection is high in solid organ transplantation (SOT) patients and often has serious consequences. Because of the lack of specificity of diarrheasymptoms cased by *Cryptosporidium* infection, it is rarely reported in patients undergoing liver transplantation (LT). It frequently delays diagnosis, coming with severe consequences. In clinical work, diagnosing *Cryptosporidium* infection in LT patients is also complex but single, and the corresponding anti-infective treatment regimen has not yet been standardized. A rare case of septic shock due to a delayed diagnosis of *Cryptosporidium* infection after LT and relevant literature are discussed in the passage.

**Case presentation:**

A patient who had received LT for two years was admitted to the hospital with diarrhea more than 20 days after eating an unclean diet. After failing treatment at a local hospital, he was admitted to Intensive Care Unit after going into septic shock. The patient presented hypovolemia due to diarrhea, which progressed to septic shock. The patient's sepsis shock was controlled after receiving multiple antibiotic combinations and fluid resuscitation. However, the persistent diarrhea, as the culprit of the patient's electrolyte disturbance, hypovolemia, and malnutrition, was unsolved. The causative agent of diarrhea, *Cryptosporidium* infection, was identified by colonoscopy, faecal antacid staining, and blood high-throughput sequencing (NGS). The patient was treated by reducing immunosuppression and Nitazoxanide (NTZ), which proved effective in this case.

**Conclusion:**

When LT patients present with diarrhea, clinicians should consider the possibility of *Cryptosporidium* infection, in addition to screening for conventional pathogens. Tests such as colonoscopy, stool antacid staining and blood NGS sequencing can help diagnose and treat of *Cryptosporidium* infection early and avoid serious consequences of delayed diagnosis. In treating *Cryptosporidium* infection in LT patients, the focus should be on the patient's immunosuppressive therapy, striking a balance between anti-immunorejection and anti-infection should be sought. Based on practical experience, NTZ therapy in combination with controlled CD4 + T cells at 100–300/mm^3^ was highly effective against *Cryptosporidium* without inducing immunorejection.

## Background

*Cryptosporidium* is a parasite transmitted directly by water, soil, and food contaminated with *Cryptosporidium* [1]. It has been recognized as a significant pathogen of diarrhea in immunocompromised hosts [2]. The symptoms of watery diarrhea result from *Cryptosporidium* infecting the microvilli layer of intestinal epithelial cells in humans and animals, causing sodium malabsorption, chloride secretion, and increased intestinal permeability in the host intestinal [3]. diarrhea caused by *Cryptosporidium* infection is self-limiting, lasting from just a few days to 14 days in immunocompetent patients. However, immunocompromised patients often manifest profuse and prolonged watery diarrhea, sometimes accompanied by nausea, vomiting, abdominal pain and fever [2, 4]. It has been shown that *Cryptosporidium* infection is high in solid organ transplantation (SOT) patients and often has serious consequences [1-3, 5-7].

With advances in surgical techniques and improved immunosuppression protocols, the overall survival rate of liver transplantation (LT) patients has improved considerably. Still, post-transplant infection remains the leading cause of death in LT patients [8]. Diarrhea is a common problem in LT patients, and results of previous studies indicate a 20–50% prevalence of diarrhea in SOT patients [9]. Usually, physicians attribute the cause of diarrhea in SOT patients to the administrating of immunosuppressive drugs and common bacterial and viral infections, rarely considering conditional pathogenic microbial infections such as *Cryptosporidium* [5]. However, in actual studies, it has been found that a large proportion of diarrhea in patients undergoing LT is caused by *Cryptosporidium* [6, 7, 9, 10]. The severe lack of vigilance and awareness of *Cryptosporidium* infection has led to a delay diagnosing diarrhea due to *Cryptosporidium* infection in LT patients and has often resulted in grave consequences such as immunorejection and shock [1-3, 5-7]. In treating *Cryptosporidium* infection in LT patients, the only approved anti-*Cryptosporidium* drug, Nitazoxanide (NTZ), is not recommended in immunodeficient and immune-rejection patient populations. Making *Cryptosporidium*-induced diarrhea a severe and under-recognized cause of diarrhea in liver patients and associated with high mortality [11-13]. *Cryptosporidium* infection in adult LT patients is rarely reported and causes more symptoms and more severe consequences in LT patients than in patients with more reported renal transplantation combined with *Cryptosporidium* infection [1, 2, 5-7, 10, 11, 14].

As we know that, there are no previous detailed case reports on the characteristics of the disease and the course of treatment following *Cryptosporidium* infection in LT patients. We describe a LT patient whose delayed diagnosis of *Cryptosporidium* infection led to severe consequences. He presented with watery diarrhea as the first manifestation and, after unsuccessful treatment at other hospitals, developed septic shock from the prolonged diarrhea with respiratory distress and impaired liver and renal function. Eventually, the patient was diagnosed with *Cryptosporidium* infection and his symptoms improved after receiving immunosuppressive adjustment and anti-*Cryptosporidium* treatment.

## Case presentation

A 55-year-old man, who received LT two years ago, developed diarrhea after an unclean diet 20 days ago and had difficulty breathing for five days. The patient's history included LT for cirrhosis two years ago and postoperative use of tacrolimus (2 tablets bid), Mycophenolate Mofetil (3 tablets bid), and sirolimus (1 tablet qd) to control immune-rejection with good results. The patient had recurrent diarrhea 20 days ago after an unclean diet, with symptoms of loose yellow stools with mucus 6–8 times a day, and did not improve after taking "berberine hydrochloride". Ten days ago, the patient's symptoms worsened, and he relieved dark green stools more than ten times a day, and gradually developed symptoms such as thirst, profoundly sunken eye sockets, palpitations and dyspnea. The patient's examination at the local hospital showed a white blood cell (WBC) count of 15. 61 × 10^9^/L, a significant decrease in potassium ions (2. 45 mmol/L), and a significant increase in creatinine level (337 umol/L). As a result, the local hospital discontinued the patient's immunosuppressive drugs and treated diarrhea and intestinal infections with Meropenem and Montmorillonite, but the results were poor. Because the patient developed symptoms such as dyspnea and oliguria, he was diagnosed with septic shock. However, after treatment with a ventilator and Continuous Renal Replacement Therapy (CRRT), diarrhea and infection were not effectively controlled, and the symptoms worsened. As a result, the patient was transferred to the Department of Intensive Care Unit of West China Hospital.

The patient was admitted with ventilator-assisted ventilation. A femoral vein placement tube for CRRT used outside the hospital was found at the root of the thigh. Patient's general condition on admission (Table 1): temperature 38. 4 °C; pulse 130 beats/min; respiratory rate: 18 breaths/min; blood pressure 100/67 mmHg (on norepinephrine 0. 4ug/kg/min). The patient had a small number of wet rales on lung auscultation, an oxygenation index (PO2/FIO2) of approximately 110, and no significant abnormalities on abdominal examination. The patient's laboratory findings showed a significant systemic inflammatory response, renal impairment and immunosuppressive state. Inflammatory indexes: white blood cell count 19. 21 × 10^9^/L; neutrophil ratio 92. 7%; CRP: 190 mg/L; interleukin 6: 336 pg/L, PCT: 3. 32 ng/L; abnormal biochemical indexes: creatinine 170umol/L; potassium: 5. 23 mmol/L; immune function: lymphocyte count: 0. 45 × 10^9^/L. The patient's abdominal CT suggested a high amount of colorectal gas stool and a dilated bowel.

**Table 1:**
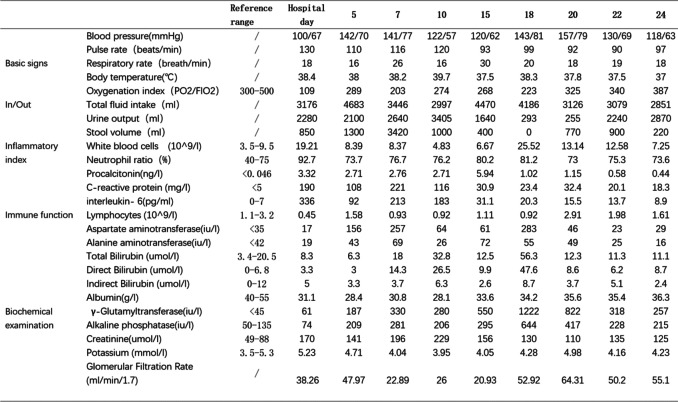
The patient’s vital signs and laboratory data during representative hospital days

Based on the patient's admission examination and the external medical records, we initially determined that the patient was in septic shock caused by infectious diarrhea with abdominal and pulmonary infections and multiorgan functional impairment. The patient was then admitted to our hospital and immediately given imipenem cystatin 1 g q8h and voriconazole 0. 2 g q12h for anti-infection. The patient's immunosuppressive drugs continued to be stopped. The patient's routine stool test was sent to identify the pathogen causing the diarrhea, but no meaningful results were obtained. On the second day, the patient's blood culture suggested Gram-positive coccus infection and chest CT suggested fungal infection. We added amphotericin B 10 mg bid and tigecycline 100 mg q12h to strengthen the anti-infection. By day 5, the patient's temperature peak was gradually reduced, and the oxygenation index rose to approximately 300. We reduced the dose of paroxysmal sedative medication discontinue the ventilator and transfer it out of the intensive care unit. As the patient's lymphocyte count rose to 1. 58 × 10^9^/L, we reintroduced him to Mycophenolate Mofetil 500 mg bid to suppress the patient's immune function.

**Fig. 1:**
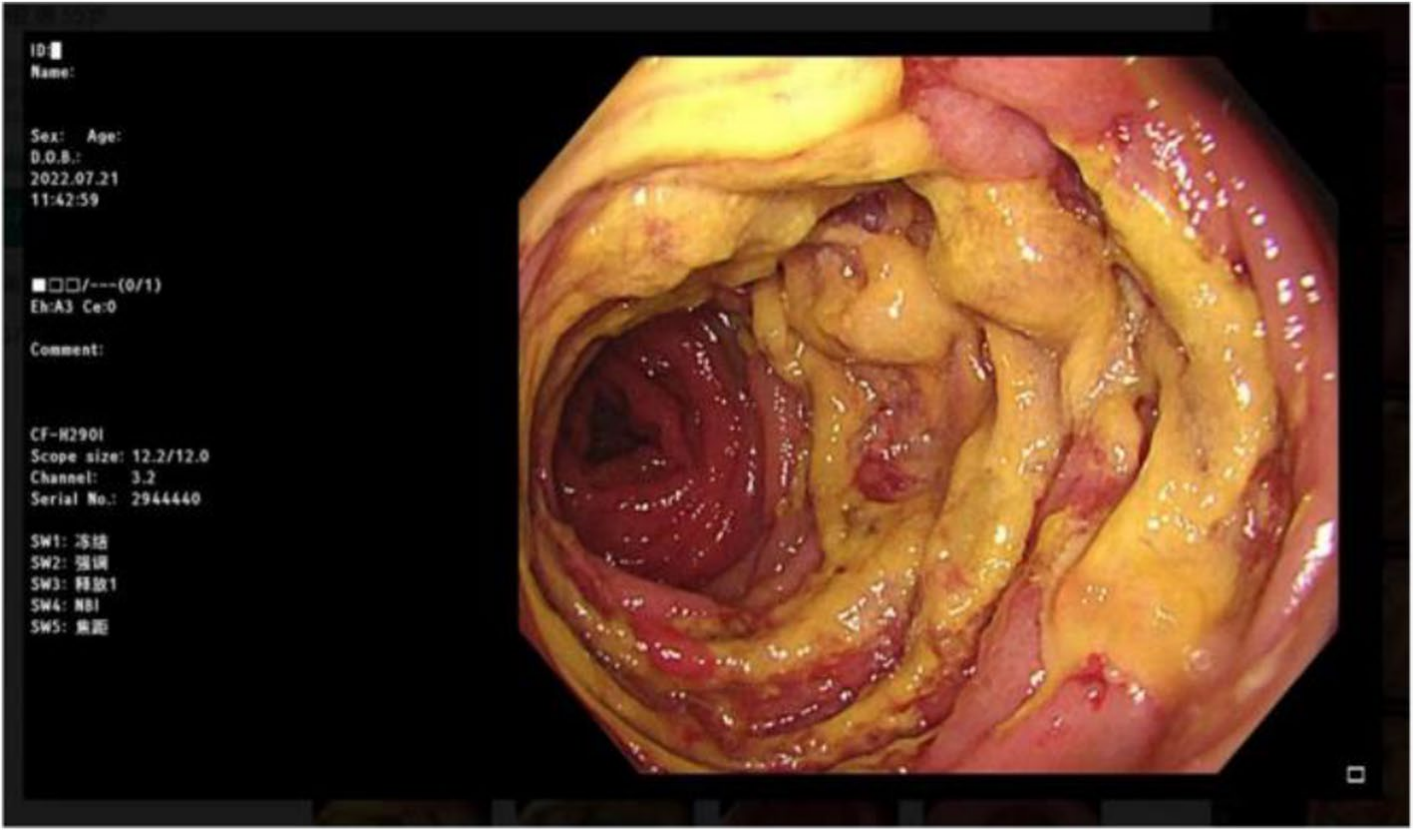
The stereoscopic presentation was segmental mucosal changes in the cecum department and ascending colon, suspicious of specific infections

**Fig. 2:**
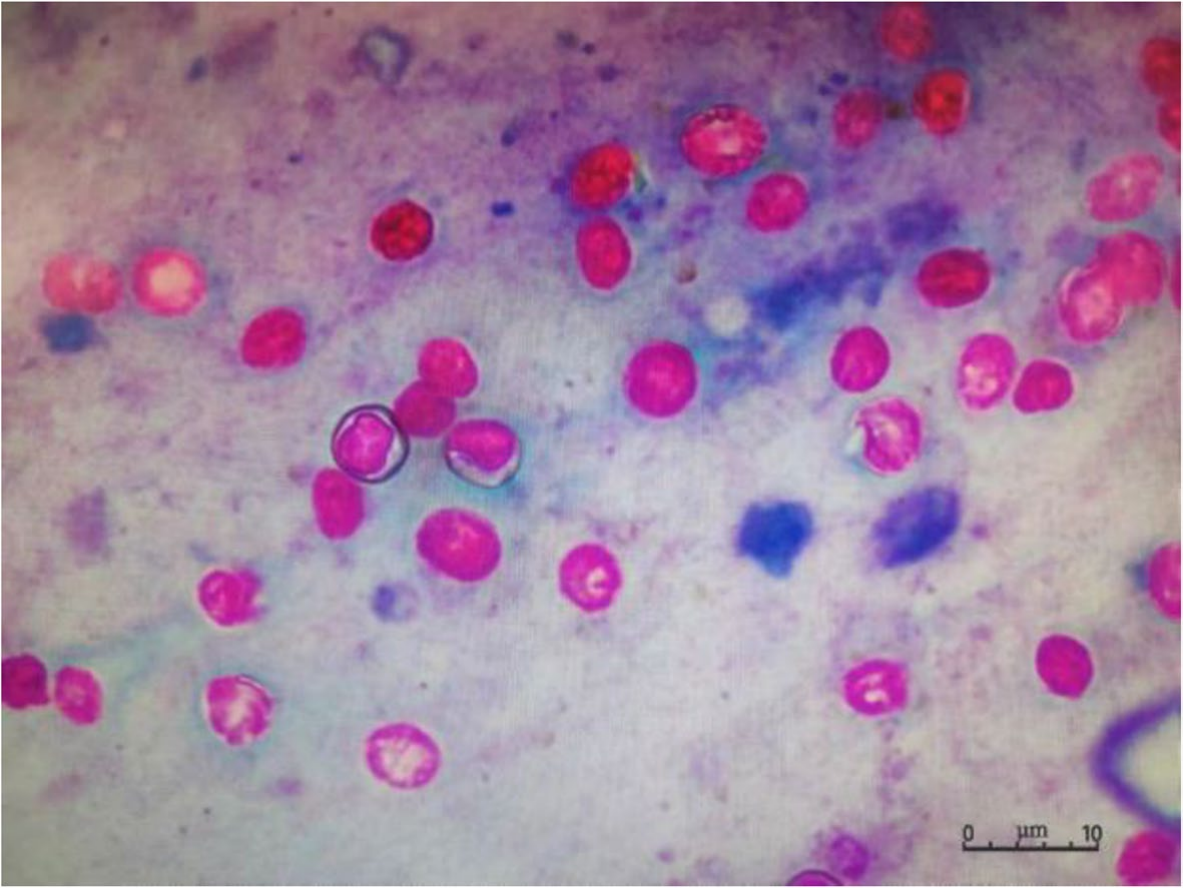
In the patient's modified antacid-stained stool specimen, the* Cryptosporidium* oocysts were rose-red with a blue-green background, the ascospores within the capsule were irregularly arranged, and the residual bodies were brown granular(× 100)

**Fig. 3:**
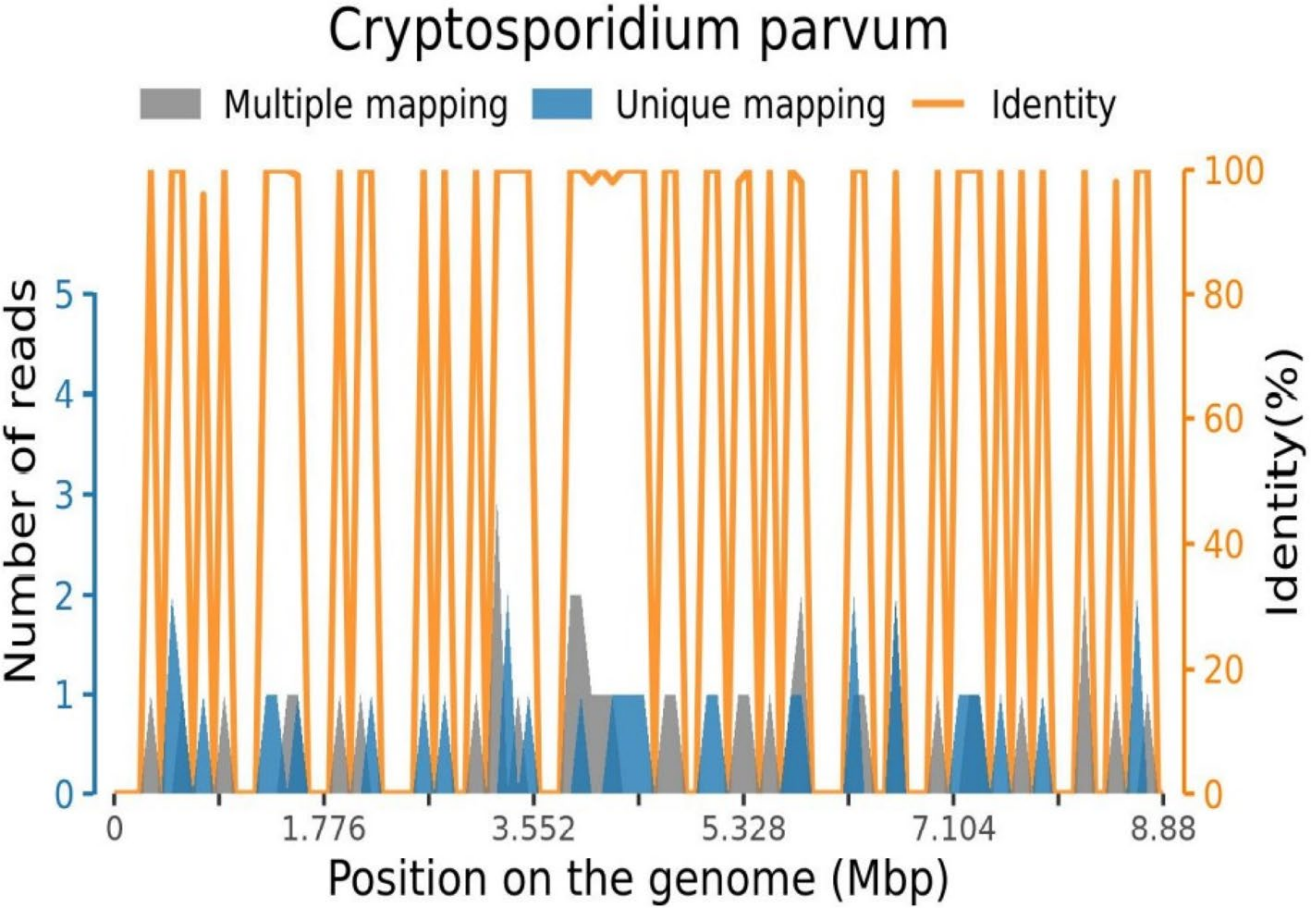
NGS detection of *Cryptosporidium* parvum

On day 7, the patient again developed massive watery diarrhea and unstable blood pressure, a decrease in oxygenation index(PO2/FIO2) to 200, increased inflammatory markers, and abnormal liver function. We considered a drug-related liver injury and parasite-associated diarrhea. So we stopped the patient's enteral nutrition, added antidiarrhea (minocycline hydrochloride) and hepatoprotective drugs (polyene phosphatidylcholine) and adjusted the antibiotic regimen to ceftazidime avibactam (2. 5 g q12h), amantadine (1000 mg q8h), voriconazole (0. 1 g q12h) and mucilage sulfate (750, 000u q12h). To clarify the aetiology of the patient's diarrhea, we performed a gastroscopy on day 8. The stereoscopic presentation was (Fig. 1): segmental mucosal changes in the cecum department and ascending colon, suspicious of specific infections. Because the patient's multiple stool tests were negative, we considered atypical pathogenic infections. Therefore, we stained the patient's stool specimen with antacid and performed high-throughput sequencing (NGS) on the blood specimen. After two days, a large number of *Cryptosporidium* was detected in the patient's stool (Fig. 2). NGS detection of *Cryptosporidium parvum* sequence number 33 with the specific information that NGS detection covers a total length of 2951 (bp) on the genome with coverage of 0. 0332% and an average depth of 1. 00X, suggesting the presence of large amounts of Cryptosporidium in the blood of this patients (Fig. 3). Furthermore, we immediately discontinued Mycophenolate Mofetil and looked for an anti-*Cryptosporidium* infection treatment option. According to previous reports, *Cryptosporidium* infection is frequently seen in children with primary immunodeficiency. *Cryptosporidium* infection causes symptoms such as sclerosing cholangitis and pulmonary *Cryptosporidium* infection in children with immunodeficiency. There was a high degree of similarity to the present case regarding both symptoms and findings. In a case report of a CD40L-deficient infant diagnosed with *Cryptosporidium* infection, after treatment with nitazoxanide and azithromycine, the patient was doing well, this report served as an important reminder for our treatment, and we immediately reviewed the instructions for NTZ [15, 16]. NTZ is the only approved anti-*Cryptosporidium* drug on the market. Because NTZ is not recommended in the instructions for use in an immunodeficient population, we used an anti-*Cryptosporidium* regimen of oral azithromycin (1000 mg tid) and alliin (500 mg tid). Five days after that, the patient showed a slight improvement in diarrhea, but liver and kidney function continued to deteriorate, and we administered continuous CRRT. On day 18, as the patient's glutamyl transpeptidase and alkaline phosphatase continued to rise, we considered *Cryptosporidium* retrograde biliary infection. To prevent the development of sclerosing cholangitis and immunorejection, we changed the anti-*Cryptosporidium* infection regimen to NTZ (500 mg tid) and allicin (500 mg tid) while continuously monitoring the patient's immune function and using cyclosporine to suppress immune function if necessary to keep CD4 + T cells were controlled at 100–300/mm^3^. The treatment plan had a good effect. The number of diarrhea gradually decreased, liver and kidney function gradually recovered, CRRT was stopped on day 22, and the patient was taken off the ventilator on day 23. On days 24 and 25, the patient underwent faecal antacid staining, and no *Cryptosporidium* was found. On day 28, the patient was transferred to the general ward and recovered well with his family. As the patient's renal function is severely compromised, we recommend that patients regularly review their renal function and pay attention to preventing infection from avoiding progression to the stage of renal failure.

## Discussion and conclusion

*Cryptosporidium* was first observed and reported by Ernest Edward Tyzzer in 1907, and the first report on human cryptosporidiosis was made in 1976. Since then, *Cryptosporidium* infections have been increasingly reported in children and immunocompromised patients, and there has been a growing recognition of *Cryptosporidium* as a seriously harmful diarrhea pathogen [17]. As the number of organ transplants skyrocketed, *Cryptosporidium* has again become an important pathogen that endangers the lives of SOT patients [3-14, 18, 11, 19, 20]. Common causes of *Cryptosporidium* infection in patients receiving immunosuppressive therapy after LT include an unclean diet, swallowing water while swimming, and lack of hand hygiene after touching pets. A high temperature and high humidity living environment also could be a high-risk factor for *Cryptosporidium* infection [2, 18]. *Cryptosporidium* infection in LT patients is characterized by:1. Watery Diarrhea lasting more than two weeks with poor response to antibiotic therapy, usually accompanied by vomiting, anorexia, crampy abdominal pain, and low-grade fever. 2. Extra-intestinal symptoms:increasing immune-rejection of the transplanted liver, increasing transaminases, symptoms associated with sclerosing cholangitis. 3. *Cryptosporidium* may not be detected in a patient's stool culture, but a typical inflammatory response can be seen by colonoscopy [2, 8, 11, 19]. *Cryptosporidium* infection causes prolonged and massive diarrhea in LT patients, increasing the likelihood of secondary infection, and liver and kidney impairment due to wasting, low sodium, and dehydration resulting in high tacrolimus concentrations [5, 8, 9, 20, 21]. We suggest that patients and clinicians should enhance their ability to recognize diarrhea in LT patients, in view of the high risk and low early recognition of *Cryptosporidium* infection in LT patients.

Diagnosis of *Cryptosporidium* infection in LT patients requires a three-pronged approach of history taking, symptomatology, and pathogenic examination. In this case, the patient's watery diarrhea dozens of times a day made us think of a bacterial infection. However, no common pathogenic bacteria were found in the routine stool examination, which could be a reason why the primary hospital underestimated the severity of the infection and delayed the diagnosis of *Cryptosporidium* infection and thus did not treat the patient aggressively. After admission to our department, as the patient's diarrhea symptoms fluctuated with the dose of immunosuppressive drugs, we supposed that the patient's symptoms might be associated with immune status. We further questioned the patient's family about the patient's medical history and learned that the patient had eaten insufficiently cooked meat prior to before becoming ill. Combined with the typical inflammatory reaction found by colonoscopy, we speculated the possibility of atypical parasitic infection. Furthermore, we found *Cryptosporidium* oocysts in fecal antacid staining and *Cryptosporidium* DNA fragments in blood NGS, which assured us of the diagnosis of *Cryptosporidium*. After effective anti-*Cryptosporidium* treatment, the patient's diarrhea symptoms were resolved, while the renal impairment due to septic shock forced the patient to undergo regular Renal Examination. For LT patients presenting with diarrhea, examination. Clinicians should simultaneously consider the possibility of *Cryptosporidium* infection during standard pathogens screening. Tests such as colonoscopy, fecal antacid staining and blood NGS sequencing can help to identify and treat *Cryptosporidium* infection early and avoid severe consequences due to delayed diagnosis [14, 18, 11, 19-23, 13, 24, 25].

Treating *Cryptosporidium* infection in LT patients, as the reported case, requires careful consideration by physicians. NTZ is the only available drug to treat *Cryptosporidium* that acts against *Cryptosporidium* by inhibiting pyruvate-ferric oxidoreductase activity and enhancing the body's immune response [2, 12, 22]. In experimental studies, it showed that gastrointestinal clearance of *Cryptosporidium* is dependent on CD4 + T cell-mediated immunity and interleukin-12-mediated production of interferon-gamma. NTZ resistance to *Cryptosporidium* also relies on normal organismal immune responses. Therefore, adjustment of immunosuppressive regimens needs to be considered first in the treatment of *Cryptosporidium* infection in LT patients, and reducing immunosuppression is first recommended [12-14, 18]. In previous trials, LT patients on cyclosporine were less likely to be infected with *Cryptosporidium* and develop graft dysfunction than patients on tacrolimus, so we discontinued the patient's immunosuppressive tacrolimus and used cyclosporine only when necessary [20, 23]. In a randomized study of NTZ in HIV-infected patients with cryptosporidiosis, the investigators observed an excellent response to NTZ in patients with CD4 + T cell counts > 50/mm^3^. As a result, we test the patients regularly for lymphocyte sorting counts and control cyclosporine dosage to control the patient's CD4 + T cells at 100–300/mm^3^ to achieve a state in which the organism responds well to *Cryptosporidium* but does not cause serious immune-rejection [20, 23, 13]. Among other possible treatment options for *Cryptosporidium* in LT patients, macrolides such as rifabutin and azithromycin have been shown to reduce *Cryptosporidium* infection potentially. In addition, oral bovine immunoglobulin (hyperimmune colostrum) appears to be a viable alternative treatment [24, 25]. Currently, fewer drugs are available for treating *Cryptosporidium* infection in LT patients, and drugs under development, such as calcium-dependent protein kinases, microtubule formation inhibitors, hexokinase, lactate dehydrogenase, inosine-5-monophosphate dehydrogenase and fatty acyl-coenzyme a binding inhibitors and anti-parasite vaccines, offer great hope for our future control of this disease [26, 27].

In conclusion, we report the case of septic shock due to delayed diagnosis of *Cryptosporidium* infection after LT. It involves the characteristics of *Cryptosporidium* of the patient and the treatment plan in terms of disease changes, examination results and treatment plan adjustments at the stage of the disease, and the patient's prognosis and regression. We emphasize that *Cryptosporidium* infection has become a serious but neglected factor affecting the survival and quality of life of LT patients in practice, and the resulting delay in diagnosis often leads to severe consequences. *Cryptosporidium* screening should be routinely performed to identify and treat *Cryptosporidium* infection early when receiving LT patients with diarrhea [3-14]. In treating *Cryptosporidium* infection in LT patients, clinicians should focus heavily on the immunosuppressive treatment of patients to find a balance between anti-immunorejection and anti-infection [10–14, 18, 11, 19–23, 13, 24–26]

## Data Availability

Data sharing is not applicable to this article as no datasets were generated or analysed.

## Abbreviations

SOT: Solid organ transplantation
LT: Liver transplantation
CT: Computed tomography
NGS: High-throughput sequencing
NTZ: Nitazoxanide
CRRT: Continuous renal replacement therapy
HIV: Human immunodeficiency virus
WBC: White blood cell
CRP: C-reactive protein
PCT: Procalcitonin
PaO2: Partial pressure of oxygen
FiO2: Fraction of inspired oxygen
